# Self-Regulated Dynamical Criticality in Human ECoG

**DOI:** 10.3389/fnint.2012.00044

**Published:** 2012-07-19

**Authors:** Guillermo Solovey, Kai J. Miller, Jeffrey G. Ojemann, Marcelo O. Magnasco, Guillermo A. Cecchi

**Affiliations:** ^1^Department of Psychology, Columbia UniversityNew York, NY, USA; ^2^Department of Physics, University of WashingtonSeattle, WA, USA; ^3^Department of Neurological Surgery, University of WashingtonSeattle, WA, USA; ^4^Laboratory of Mathematical Physics, The Rockefeller UniversityNew York, NY, USA; ^5^IBM Research, T. J. Watson LaboratoryYorktown Heights, New York, NY, USA

**Keywords:** electrocorticography, criticality, motor movement

## Abstract

Mounting experimental and theoretical results indicate that neural systems are poised near a critical state. In human subjects, however, most evidence comes from functional MRI studies, an indirect measurement of neuronal activity with poor temporal resolution. Electrocorticography (ECoG) provides a unique window into human brain activity: each electrode records, with high temporal resolution, the activity resulting from the sum of the local field potentials of ∼10^5^ neurons. We show that the human brain ECoG recordings display features of self-regulated *dynamical* criticality: dynamical modes of activation drift around the critical stability threshold, moving in and out of the unstable region and equilibrating the global dynamical state at a very fast time scale. Moreover, the analysis also reveals differences between the resting state and a motor task, associated with increased stability of a fraction of the dynamical modes.

## 1 Introduction

The regulation of excitation and inhibition is an essential feature of neural systems. Most theoretical and experimental explorations rely on a static analysis of the balance of inhibitory and excitatory inputs, or emphasize the attainment of this balance through slow plastic changes (Song et al., 2000; Froemke et al., 2007; Gandhi et al., 2008). The theory of self-regulated criticality, however, stipulates that in order to fully realize its expressive potential, a complex system must constantly, dynamically poise itself at the critical point, i.e., at the boundary between stability and instability (Bak et al., 1987), so that it can be maximally responsive to its input. In the case of neural systems, the changing bombardment of inputs additionally requires that this balance be attained at short time scales, lest the overall activity becomes unstable or muted while the system slowly adjusts itself.

Criticality is usually understood in a purely statistical sense, i.e., by identifying anomalous, heavy-tailed distributions (as opposed to Gaussian or Poisson) of the relevant physiological or neuroanatomical variables. There are indeed many examples of statistical criticality in experimental neuroscience (Chialvo, 2010; Mora and Bialek, 2011): neuronal avalanches (Beggs and Plenz, 2003; Haldeman and Beggs, 2005; Gireesh and Plenz, 2008; Friedman et al., 2012), spiking correlations in the retina (Schneidman et al., 2006; Hennig et al., 2009), and inter-area activation in functional imaging (Eguíluz et al., 2005; Fraiman et al., 2009).

Nevertheless, neurobiology and biology at large abound with examples of systems that are *dynamically critical*, i.e., that regulate their dynamics constantly and rapidly, while they reside at the critical point. These systems include line attractors in motor control (Seung, 1998; Seung et al., 2000) and decision making (Machens et al., 2005), self-tuned Hopf bifurcations in the auditory periphery (Choe et al., 1998; Camalet, 2000) and olfactory system (Freeman and Holmes, 2005), as well as models of regulated brain criticality (Bienenstock and Lehmann, 1999). Dynamical criticality, however, has not been characterized for large, extended neural systems. It is not evident that such systems should display this behavior, as critical dynamics may only be regulated locally, or conversely only be revealed in large enough networks (Ohiorhenuan et al., 2010). For this purpose, we used electrocorticography (ECoG) recordings, a technique that provides a unique window into human brain activity (Miller et al., 2009a; Ritaccio et al., 2010). Recently, Raichle and colleagues have shown that ECoG signals display power-law statistics in the spectral content of the electrical potential time series (He et al., 2010), although a different study found power-law scaling without evidence for criticality (Miller et al., 2009a). Such behavior of the ECoG voltage time series would be an indication of a system in a critical state, but does not provide further insights as to possible mechanistic interpretations or functional implications, and moreover, it does not imply that the system is dynamically critical.

In order to explicitly estimate the properties of large-scale brain physiology vis-a-vis dynamical stability, we analyzed large-scale human ECoG array recordings, fitting the data to auto-regressive models. As we demonstrate in the next sections, these recordings exhibit robust and generic dynamical as well as statistical criticality. The regulation of dynamical modes of activation takes place at a fast time scale, resulting in the modes drifting around the critical threshold as they move in and out of the unstable region. Moreover, the analysis also reveals differences between the resting state and a motor task, associated with increased stability of a fraction of the dynamical modes.

## 2 Materials and Methods

### 2.1 Experimental data

We analyzed ECoG recordings from 11 human subjects. The experiments were performed with epilepsy patients at Harborview Hospital in Seattle, WA (USA), according to an IRB approved by the University of Washington. We provide here only a brief description of the experimental procedure as the details have been described previously (Miller et al., 2009c). ECoG potentials were recorded from subdural electrode grids. Each electrode in the array had a ∼5 mm^2^ platinum surface exposed to the subdural brain surface. The grids [48 electrodes (6-by-8 array) in 2 patients and 64 (8-by-8 array) in the rest] were implanted for extended clinical monitoring and localization of seizure foci. Cortical potentials were recorded at a sampling frequency *F_s_* = 1000 Hz, with respect to scalp reference and ground. The subjects performed a finger-movement task. They were cued with a word displayed on a bedside monitor to move fingers independently during 2 s movement trials. They typically moved a finger 3–5 times during each trial, but some trials included many more movements. A 2 s rest trial (blank screen) followed each movement trial. There were 30 movement cues for each finger, and trial types were interleaved randomly.

The ECoG signals were notch filtered at 60, 120, and 180 Hz and then band-pass filtered (5–200 Hz) using a 6th-order Butterworth filter.

### 2.2 Data analysis

#### 2.2.1 Auto-regressive model and eigenmode estimation

Properties of a natural system can be inferred from stochastic time series models fitted to the experimental observations. Auto-regressive models are one such family of models that have been used in many different areas of natural and social science research. They can be used to characterize oscillatory patterns and to make predictions in complex systems. An auto-regressive model for the variable *y* is defined as $y\left(n\right)=\Sigma_{i=1}^{p}a_{i}y\left(n-i\right)+e\left(n\right),\;$ where *n* indicates the discrete time step of the series, *p* is the order of the model, *a_i_* are the constant coefficient parameters, and *e* is a noise term. In our case, we wish to model the joint recordings of all electrodes, a multivariate time series. Therefore, the variables are now represented by the column vector **y**, the coefficient parameters **a***_i_* are matrices, and the error vector **e** is a column vector. The state of each variable depends on the previous values of all variables (the evolution of each component can be correlated), in a manner described by **a***_i_*. Given an experimentally observed time series, it is possible to fit the parameters of the auto-regressive model to it. By doing so, the dynamics of the observed time series is then *encoded* in the parameters of the model.

In this work we use auto-regressive models to describe the dynamics of a highly non-linear system as the human brain. A single auto-regressive model of any order would not, by definition, describe the entire time series of ECoG potentials for the whole experiment (10 min). Therefore, we use auto-regressive models of order 1, AR(1), in relatively short time windows, within which the system is assumed to be linear. The parameters of the model (the coefficients of the matrix **A** = **a**_1_) are fitted locally in time to **V**(*n*), the ECoG potentials time series. From each local AR(1) model we derive the associated eigenmodes and their stability parameter, defined as the absolute value of the eigenvalues of *A*. **V**(*n*) is a *N_e_*-dimension column vector, where *N_e_* is the number of electrodes and *n* is the time step number (*n* = *t* × *F_s_* where *t*). To estimate the eigenmodes at time *t* = *n*/*F_s_* we first isolate a 250 ms window of **V** around *t*. This is the *local* time series to which we will fit an AR(1) model, defined by:

(1) $$x_{m+1}=A\left(t\right)x_{m}+e_{m}.$$

In equation (1), **x** is the time series variable that evolves in discretized time steps *m*, **A**(*t*) is the *N_e_* × *N_e_* coefficient matrix and **e** is an uncorrelated noise vector. We estimate **A** for each time step *n* using the algorithm of Neumaier and Schneider (2001) on overlapping windows shifted by 1 ms. Basically, an estimation of the matrix **A** is obtained by casting the AR(1) model in the form of an ordinary regression model and then estimating the parameters of the regression model using a standard least square method. In the Supplementary Material, we show that this procedure extracts the expected eigenmodes of a well known critical dynamical system undergoing a Hopf bifurcation.

The temporal dynamics of an AR(1) model is determined by the eigenmodes of **A** (with eigenvalues λ = |λ|*e*^*i* argλ^). If |λ| < 1 the mode is stable and if |λ| > 1 the mode is unstable. Stable modes tend to damp and unstable modes tend to explode (exponentially). Complex eigenvalues define a frequency of oscillation (*f*_λ_ = *F_s_*|argλ|/(2π)) of the eigenmode. We use the convention according to which −π < argλ < π, to ensure that a pair of complex conjugate eigenvalues is associated with a single frequency.

#### 2.2.2 Avalanches

The ECoG potentials **V**(*n*) were converted to binary strings **s**(*n*), so that each component of **s**(*n*) describes the activity pattern in time of an individual electrode. If the absolute value of the potential for an electrode is larger than a threshold at time step *n* (|*V_i_*(*n*)| > *V_th_*), the string is deemed *active* and *s_i_*(*n*) = 1. Otherwise, *s_i_*(*n*) = 0. Figure 3A illustrate the conversion from ECoG potential to *s* on a single channel. We set the threshold at *V*_th_ = 3.5 SD, where SD is the average standard deviation of *V_i_*. However, statistical criticality is robust with respect to changes in the threshold, as shown in Figure S2 in Supplementary Material. A raster plot (Figure 3B-bottom) is a representation of the binary strings as a function of time. The number of active electrodes at a given time is *N_act_*.

An avalanche is defined as an event that occurs when any *N_act_* > 0. The size of the avalanche is defined as the sum across electrodes of *N_act_* during the avalanche. The pair-wise correlation coefficient between all pairs of electrodes is computed using the binary string version of ECoG potentials (Schneidman et al., 2006).

To reveal statistical criticality features from the data, we fit the distribution of the number of active electrodes and the size of avalanches to a discrete power-law distribution using the methods described in (Clauset et al., 2009). A power-law distribution for a discrete random variable *x* is

(2) $$p\left(x\right)=Cx^{-\alpha }\;\text{for}\;x\geq x_{min}$$

where *C* is a normalization constant. A maximum likelihood method is used to estimate the scaling parameter α and the lower bound *x_min_*.

The power-law form of a distribution can be visualized as a straight line in a log–log plot of the histogram. However, these plots are usually noisy at the right-hand end of the distribution because of sampling errors. An advantageous method of plotting the data is to calculate a cumulative distribution function (Newman, 2005). In this case, instead of plotting a histogram, we plot the probability *P*(*x*) that *x* has a value greater than or equal to *x*:

(3) $$P\left(x\right)=\int_{x}^{\infty }p\left(x^{\prime }\right)dx^{\prime }\;$$

If *p*(*x*) is power law, then the cumulative distribution function *P*(*x*) also follows a power law. Interestingly, *P*(*x*) is simply proportional to the rank of *x* (Newman, 2005). This means that to make a plot of *P*(*x*) we first sort the data in decreasing order of frequency, number them starting from 1, and then plot their ranks as a function of their frequency. All cumulative plots in this paper were made in this way (Figures 3D,E and 4 and in Supplementary Material).

### 2.3 Simulations

Two simulated signals were generated to validate the analysis and the conclusions we derived from the data: a white noise process and a Wiener process (random walk). In the white noise case, the signal of each channel is independent and draw from a Normal Distribution (mean = 0, SD = 1). In the case of a Wiener process, the signal of each channel is obtained by integrating the white noise signal, so that the Wiener signal at each channel is $V_{i}^{Wiener}\left(n+1\right)=V_{i}^{Wiener}\left(n\right)+\psi_{i},$ where ψ*_i_* is a normal distributed random number (mean = 0, SD = 1). The important difference between both simulated signals is in the power spectrum. White noise has a flat spectrum across frequencies while a random walk generates noise with a *S* ∼ 1/*f* power-law spectrum (see, for example Miller et al., 2009a).

## 3 Results

**Figure 1:**
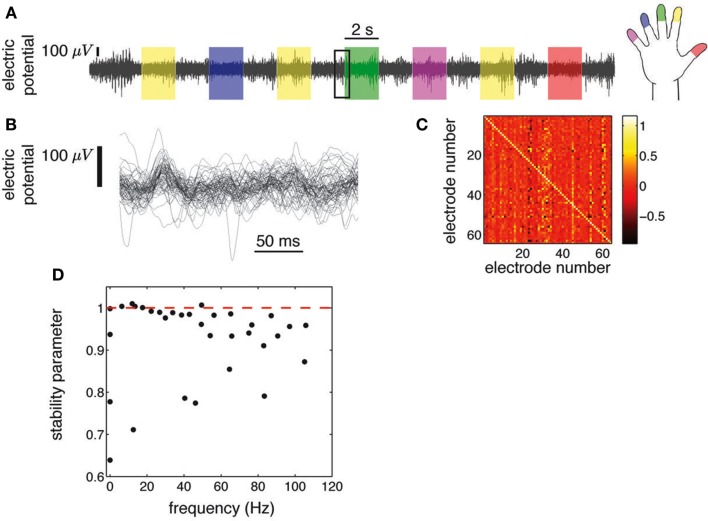
**ECoG potentials were registered at 1 kHz during 10 min using grids of 48 or 64 electrodes (Miller et al., 2009c)**. **(A)** we show the ECoG potential of one electrode during 28 s. The color shading indicates periods in which subjects were cued to move an individual finger. Cues were presented in blocks of 2 s separated by 2 s blank screen periods. **(B)** Zoom-in corresponding to 250 ms [rectangular area in **(A)**], showing all 64 electrodes. **(C)** Regression matrix corresponding to the AR(1) model fitted to the time series shown in **(B)**. **(D)** Eigenvalues of the matrix shown in **(C)**. Each complex eigenvalue is characterized by a frequency and a stability parameter (absolute value).

**Figure 2:**
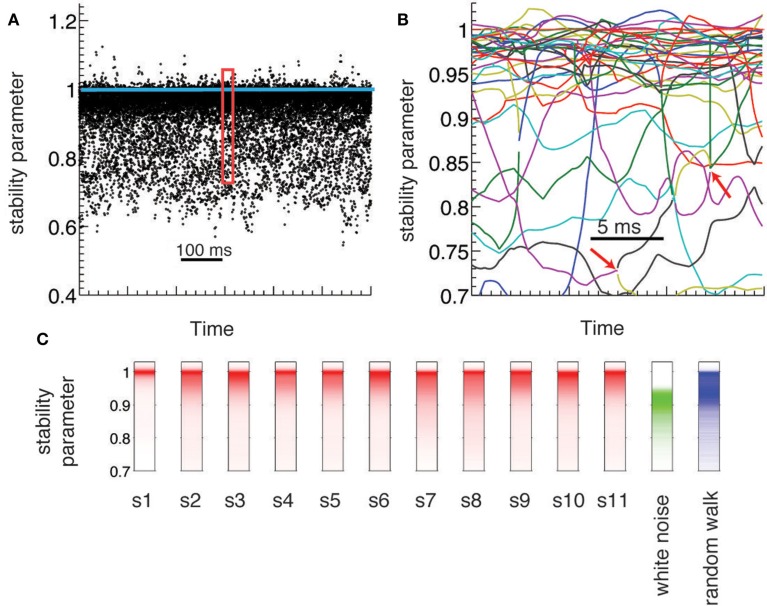
**Dynamical criticality in human brain ECoG potentials. (A)** Absolute value of the eigenmodes (stability parameter) derived from the estimated AR(1) models, plotted during 600 ms (black dots). The eigenvalues are concentrated close to the critical value (blue line), an evidence of dynamical criticality. **(B)** Zoom-in of the data A on a 30 ms window. Each colored line corresponds to the evolution of a different mode. Red arrows indicate bifurcation points: a complex mode gives rise to two real new born modes (and vice versa). **(C**) Histogram of the stability parameter for all subjects (red), and for two synthetic data (white noise and random walk).

**Figure 3:**
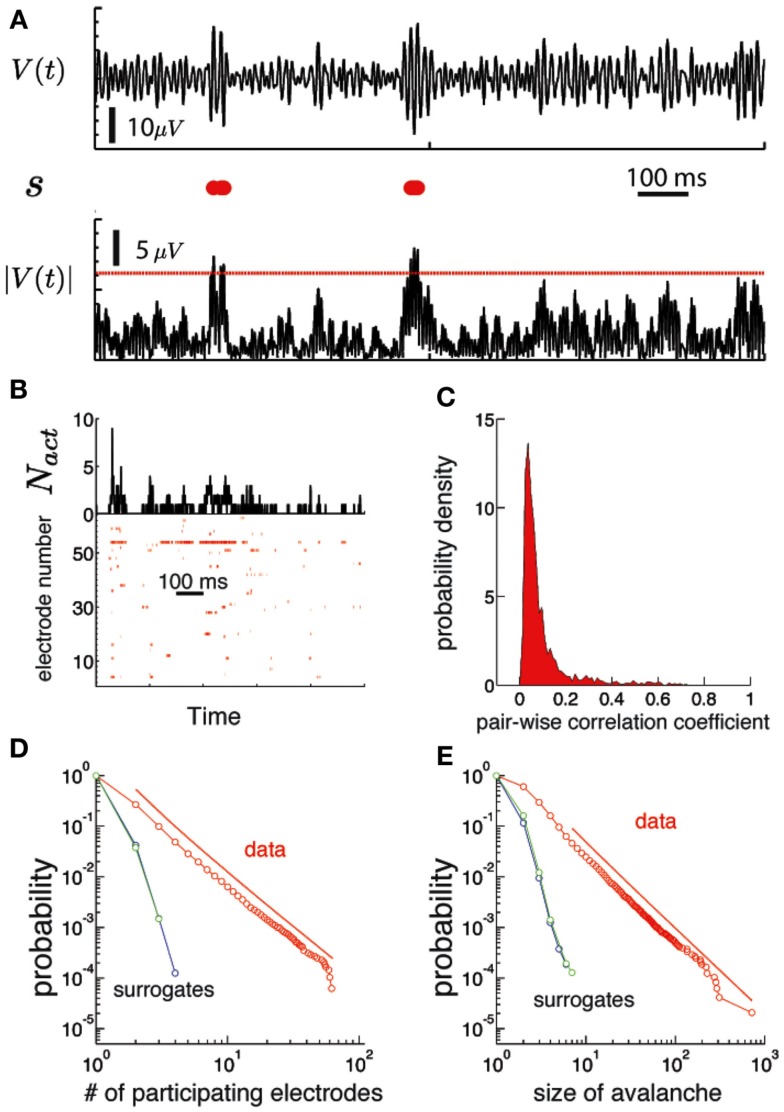
**Human brain ECoG potentials are statistically critical**. **(A)** The procedure used to obtain the binary version of the ECoG potential avalanches is illustrated for one channel. On the top we show the trace of the electric potential at one channel, *V*(*t*) as a function of time during 1 s. The lower black trace is the absolute value of the potential |*V*|. We use a threshold *V_th_* (red line) to convert this signal into a binary string *s*, displayed in red in the middle row. Each red dot correspond to a time step of the electric potential where |*V*| ≥ *V_th_* **(B)** bottom: Raster plot of a discretized version of the ECoG potential for an individual subject. Each red dot indicate an active electrode: i.e., the absolute value of the ECoG potential for that electrode (ordinate) is above a threshold at that time (abscissa). The threshold was set to 3.5 SD, where SD is the mean standard deviation, averaged over all electrodes. Top: the number of active electrodes (*N_act_*) as a function of time. An avalanche of activity is any activation event separated by regions of *N_act_* = 0. **(C)** Histogram of the linear pair-wise correlation coefficient (Pearson) calculated between all pairs of binary sequences corresponding to the activation of each electrode. **(D)** The number of unique electrodes that participate in the avalanche (1,…, *N_e_*) are power law distributed (red). The red line is a power-law fit of the data (shifted for clarity). Both simulated signals do not show a power-law distribution. **(E)** The size of the avalanches is also power law distributed (see Figure 4 to see the results for all subjects).

**Figure 4:**
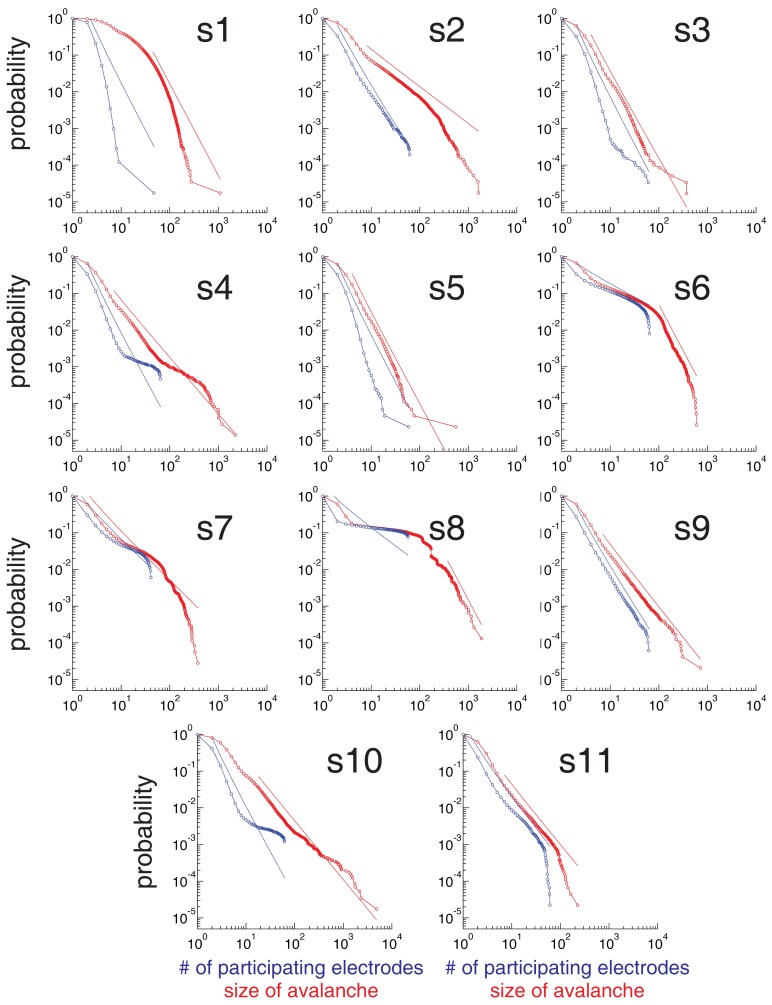
**Statistical criticality is ubiquitous among subjects**. Cumulative distribution plots of the number of electrodes that participate in an avalanche (blue) and the size of the avalanche (red) for every subject subject. Although there are differences among subjects, all distributions are long-tailed and power law in a certain range (indicated with dotted lines).

**Figure 5:**
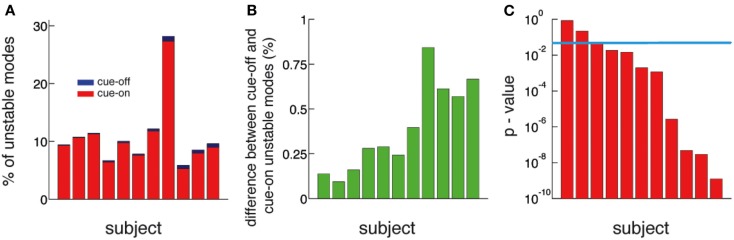
**The number of unstable modes is larger in cue-off than in cue-on periods for all subjects**. **(A)** Relative difference (%) between the mean number of unstable modes in cue-on and cue-off periods for all subjects. The blue bars correspond to the% of unstable modes in the cue-off condition and superimposed red bars correspond to the cue-on condition. **(B)** Difference between the % of unstable nodes in cue-off and cue-on conditions. This quantity is always positive, therefore there is always more unstable modes in cue-off than in cue-on periods. **(C)** The differences in the distribution of unstable modes is significant in 8 of 11 subjects. (*p* < 0.05, Kolmogorov–Smirnov test, blue line: *p* = 0.05).

**Figure 6:**
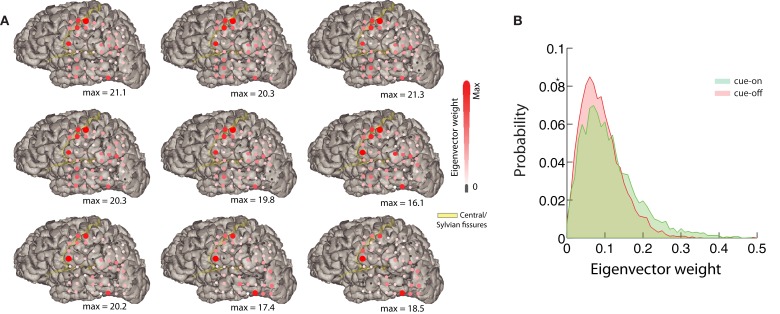
**Mapping of the eigenvectors**. **(A)** Relative difference between the mean eigenvector of cue-on and cue-off periods corresponding to the 9 most unstable modes for one subject (top left: most unstable; decreasing order from left to right, top to bottom). Each red circle corresponds to a component of the eigenvector. Its size and color represents the magnitude of the difference between conditions (eigenvector weight). The maximum difference is indicated below each subplot. The position of the red circles correspond to the position on the brain of each electrode of the array for this subject. **(B)** We show the eigenvector weight distribution for most unstable eigenvector and the component with larger difference between conditions.

The results illustrated in Figures 1 and 2, and Figures 3 and 4, provide evidence that human ECoG potentials are dynamical and statistically critical, respectively. Figures 5 and 6 illustrate how our analysis can be used to distinguish between rest and task related activity during a finger-movement task.

### 3.1 Dynamical criticality

ECoG electric potentials typically vary with an amplitude in the range of tens of μV. In Figure 1A we show the trace of an ECoG potential recorded from one electrode during 28 s. Within this period, subjects were cued to move different fingers in 2 s blocks (shaded areas) separated by 2 s of rest. Figure 1B displays the time evolution of the potential of all 64 electrodes on a window of 250 ms; this time series is fitted with an AR(1) whose regression matrix is shown in Figure 1C. The eigenvalues of this matrix are shown in Figure 1D as complex numbers, with the absolute value corresponding to the stability parameter and the phase to the frequency; the eigenvalues tend to crowd near the critical line (red dashed line), even for higher frequencies. This is further appreciated in Figure 2A, where the evolution of the eigenvalues is plotted over 600 ms, showing also how there are transient crossings above criticality (blue line). Figure 2B is a zoom-in on a 30 ms window, where eigenmodes were tracked individually, in order to display the finer temporal structure of their evolution (see in Supplementary Material). The figure shows how the eigenmodes drift in and out of the criticality zone, with their own dynamics; the instabilities thus created are not long-lived because the system does not explode in finite time.

To understand the significance of the crowding effect of the eigenvalues, we compared their distribution in the experimental data against two simulated signals: a white noise process and a Wiener noise process. In the case of the white noise, the frequency spectrum is uniform, while for the Wiener process and ECoG electric potentials the power spectral density has a power-law form (Miller et al., 2009a). The red trace in Figure 2C shows the histogram of eigenvalues for the original data over an entire experiment (10 min) for all subjects. The green trace corresponds to the eigenvalues reconstructed for a white noise signal, showing that they are distributed over a wide but practically non-overlapping range with respect to the original data. This is expected, given that there are no temporal correlations in the white noise simulated signals. Finally, the blue trace corresponds to the Wiener noise signal. Given its definition, these simulated signals are expected to display more temporal structure than white noise. Indeed, the eigenvalues histogram has a peak near |λ| = 1 as the original data; however, they do not show crowding close to the critical line, as the distribution is much wider.

### 3.2 Statistical criticality

We observed that ECoG potentials display statistical criticality, as presented in Figure 3. The upper plot in Figure 3B shows the number of active electrodes, *N_act_* during 1 s for a single human subject. Figure 3B (bottom) shows a raster plot: each row represents the activity of an electrode and a red dot indicate the time steps where the electrode is active. Figure 3C shows that pair-wise correlation between electrodes is relatively weak, with mode and mean less than 0.1. While weak, the correlation is stronger than that expected for a random sequence, and has a long tail. In fact, as shown in Figures 3D,E, the data are statistically critical. In Figure 3D, we plot the cumulative distributions of the number of electrodes that participate in an avalanche and in Figure 3E, the cumulative distribution of the size of the avalanche (e.g., the sum over *N_act_*). Both distributions are power law, with exponents 3.1 and 2.7. In contrast, the white noise and Wiener noise signals do not show statistical criticality, demonstrating that the network cooperative effects are significant. Statistical criticality is ubiquitous among subjects (Figure 4). Although there are differences among subjects, all distributions are long-tailed and power law in a certain range. The red and blue dotted lines are power-law functions with the fitted exponent for the distribution of the size of the avalanche and the number of participating electrodes, respectively. The range in which the distribution is power law is illustrated by the range of the dotted line. In Table S1 in Supplementary Material we present a summary of the results for all subjects.

### 3.3 Criticality and function

The functional implication of dynamic criticality is based on the concept that a system close to instability is more readily excited, or susceptible to perturbations (inputs), than a stable one. To demonstrate a functional role of criticality, we compared periods of rest (cue-off) with periods where the subjects were cued with a word at the screen indicating the finger they had to move (cue-on). We compare them by calculating the number of unstable modes (*N_u_*), i.e., modes whose eigenvalues exceed the critical line. We found that *N_u_* is larger in cue-off than in cue-on periods in all subjects (Figures 5A,B). The difference is small in magnitude (the relative difference is shown in Figure 5A) but significant (*p* < 0.05 in 9 of 11 subjects) as shown in Figure 5C. Given that the mean and the variance of the raw potential does not have any significant difference between cue-on and cue-off periods (see in Supplementary Material), we argue that the differences are based only on a change in the dynamical criticality of the system.

In contrast to what we observe with the dynamical behavior between cue-on and cue-off conditions, power-law distributions (characteristic of statistical criticality) does not seem to be discriminative. While we found a difference in the total number of active units between conditions for two subjects, this is not consistent across the data set. Moreover, the two conditions show similar distributions for the size of avalanches and the number of active electrodes (in Supplementary Material).

The dynamical approach can provide further functional insights. We depict in Figure 6 the result of analyzing the spatial support of the eigenvectors, i.e., the absolute value of the vector’s components laid on the two-dimensional ECoG grid. Figure 6A depicts, for the 9 more unstable vectors, the relative difference between the average for cue-on and cue-off conditions. This analysis shows that the differences are sparse and spatially structured, focused in sensorimotor and visual areas covered by the ECoG array, which emphasizes the spatiotemporal nature of the brain interactions involving a relatively simple task. The differences between the conditions can be measured statistically. This is represented in Figure 6B which shows a histogram of the eigenvector weight for one particular electrode of the most unstable mode, for both conditions (*p* < 10^−5^, KS-test).

## 4 Discussion

Structural stability of systems has been a basic tenet of non-linear dynamics theory: the qualitative behavior (defined, for instance, by an attracting fixed point or limit cycle) should not change upon small perturbations; reciprocally, transitions between different dynamical states should be exceptional (Guckenheimer and Holmes, 1983). This concept has deeply influenced systems neuroscience: one of the dominant paradigms is attractor neural networks, in which computation is *defined* by the presence of structurally stable fixed points, so much so that these are the only states where proper computation takes place (Amit, 1992). Similarly, the theory of oscillatory neuronal ensembles implies that brain computation is carried out by a limited number of structurally stable modes so that, for instance, synchronization can be attained in the γ-band, regardless of potential events taking place in the rest of the spectrum (Rodriguez et al., 1999). From a computational point of view, however, this framework is too limited, as it constraints dramatically the number of accessible states, and precludes the possibility of implementing computations with transient states, such as those produced by systems based on heteroclinic orbits (Mazor and Laurent, 2005).

In the past decade, the use of ECoG recordings to understand brain function has increased enormously, providing unique insights to a large variety of human cognitive processes (Jacobs and Kahana, 2010; Ritaccio et al., 2010). The main reason for the success of ECoG recordings is that they measure human brain activity with higher spatial and temporal resolution than any other recording technique. An example especially relevant for the present study is Miller et al. (2009c); the authors report individual digit representation in adjacent ECoG electrodes (Miller et al., 2009c), separated by 6 mm, speaking by itself of the high spatial resolution of ECoG. This observation has a direct implication for our finding, consistent with previously published reports (He et al., 2010), of statistical criticality in ECoG. One possible simple explanation for the presence of avalanches is that electrodes pick up the activity of static and isolated common sources, perhaps at different cortical depths, as opposed to a collective process of critically regulated activity. While this null hypothesis cannot be completely ruled out given the limitations of current recording techniques, the spatial task-specificity of ECoG suggests that it is unlikely. This is confirmed by our own results: the power-law distribution of the number of participating electrodes in an avalanche implies that there is a finite probability that an avalanche will involve, for instance, half of all electrodes (Figure 3D, *p* ~ 10^−3^). Given the restricted spatial extent of the eigenvectors associated with the task (Figure 6A), we find the criticality hypothesis more plausible.

The dynamical criticality we observe in ECoG recordings implies a balance between the stability of the system and its susceptibility to internally or externally induced changes. In neuro-dynamical terms, the functional advantage afforded by criticality is easily understood. The modes represent the coordinated activity of a large, distributed ensemble of neurons. Generalized instability in these ensembles is undesirable, but a highly stable ensemble would require a correspondingly strong perturbation to be modulated, and therefore be refractory to change. Under a local linear approximation, the eigenvalues of the activation modes, while drifting, remain crowded near the critical line. This implies that the brain has a mechanism to actively constrain these modes upon the effect of external (i.e., sensorial input) and internal (e.g., plasticity) agents. In this respect, the finding that the fixation condition is closer to dynamical criticality than the task may be related to the activity of the default mode network (Raichle et al., 2001; Miller et al., 2009b). This finding is consistent with the idea that brain activity is dominated by the superposition of a vast number of ongoing processes, such as planning, memory encoding and re-evaluation, and self-monitoring; in this interpretation, sensorimotor inputs and outputs, and even more demanding cognitive tasks, are “ripples in a pond” (Arieli et al., 1996), producing changes that are relatively small and fleeting, and contingent upon brain state. In the broad sense, given the complexity and dimensionality of the brain as a dynamical system, this must be true. However, the dominant paradigm of the brain as a reactive system, concerned mainly with representing the external world, will be hard to relinquish until a new formal theory is developed.

The differences we find between the more unstable resting state and the more stable task are consistently small, yet statistically significant. This is reasonable to expect, in light of the “ripples” hypothesis: while physically sizable, the finger task is computationally simple. As a proper task, it should also engage, however briefly, a relatively stable activation mode, corresponding to the observed periodic finger movement. Moreover, we find that the dynamical modulation induced by the task is distributed over a large area of the brain. This finding can be similarly interpreted: while neurons encoding the movement of individual fingers may be relatively localized, the dependences of the specific motor pattern to be executed on several physical constraints (the position of the arm relative to the body, whether the arm lies on a flat surface, etc.) and cognitive states (the task paradigm, willingness, volition, etc.), implies that task engagement is much more sophisticated than merely the end effect of movement production in a few muscles.

The evidence of statistical criticality in the size and number of sites participating in avalanches is consistent with previous findings in slice preparations (Beggs and Plenz, 2003; Haldeman and Beggs, 2005; Gireesh and Plenz, 2008) and may or may not be present in the power spectrum of the voltage time series of ECoG recordings (Miller et al., 2009a; He et al., 2010). We found the differences between task conditions in the distribution of avalanche sizes to be insignificant, in contrast to their dynamical stability, a finding that supports our claim that criticality in the brain must be considered in a broader sense. This also provides insights for the understanding of the relationship between event-related potentials (ERPs) and criticality: while it may be possible to think of ERPs as particularly large avalanches, and conversely of avalanches as a succession of internally and externally triggered ERPs, our finding rules out a simplistic explanation of statistical criticality as a direct consequence of ERPs elicited by the task.

Dynamical criticality, in contrast, has not yet been described in the spatial correlations of high temporal resolution, spatially distributed, brain array recordings like ECoG, EEG, or multi-electrode recordings. Moreover, the relationship between statistical and dynamical criticality remains unclear, and the two are often considered manifestations of the same phenomenon. But this may not necessarily be so: perfectly deterministic and stable dynamical process acting on a topologically critical tree (i.e., with a scale-free distribution of the number of links per node) can nevertheless generate a critical activation distribution. This example is particularly relevant for neural systems, given the experimental evidence of criticality in the topology of neural circuits (Ma’Ayan et al., 2008), and the theoretical demonstration that synaptic time-dependent plasticity can create these topologies (Fiete et al., 2010; Kozloski and Cecchi, 2010).

One the reasons hindering our understanding of criticality is that most models of statistical criticality lack smooth dynamics and therefore resist analysis in terms of the qualitative theory of dynamical systems, and such qualitative theory is well-suited for the description of dynamical criticality. The network model presented in Magnasco et al. (2009), based on anti-Hebbian synaptic plasticity (Destexhe and Marder, 2004; Lamsa et al., 2007), provides a possible bridge between these two notions of criticality, and reproduces qualitatively the critical behavior of the ECoG recordings. In this model, a network spontaneously poises itself at a dynamically critical state by maintaining all its eigenvalues hovering around the critical line; occasional excursions into instability of some modes create avalanche-like bursts of activity, which are themselves distributed as a power law. The qualitative agreement between the Magnasco et al. network model and our present empirical finding is encouraging, and provides a foundation for further exploration of widespread cortical dynamics.

## Author Summary

We show that human brain ECoG recordings display features of self-regulated dynamical criticality: dynamical modes of activation drift around the critical stability threshold, moving in and out of the unstable region, and equilibrating the global dynamical state at a very fast time scale. Moreover, the analysis also reveals differences between the resting state and a motor task, the later associated with increased stability of a fraction of the activation modes.

## Conflict of Interest Statement

The authors declare that the research was conducted in the absence of any commercial or financial relationships that could be construed as a potential conflict of interest.

## Supplementary Material

The Supplementary Material for this article can be found online at: http://www.frontiersin.org/Integrative_Neuroscience/10.3389/fnint.2012.00044/abstract
